# Neutrophil percentage-to-albumin ratio as a novel hematologic biomarker for predicting arteriogenic erectile dysfunction

**DOI:** 10.3389/fendo.2025.1729618

**Published:** 2026-01-19

**Authors:** Yangyang Mei, Guoyang Zhang, Yu Liu, Yiming Chen, Renfang Xu, Hao Lu, Hui Gao, Xianchao Sun, Wei Zhang, Xingliang Feng, Qianfeng Zhuang

**Affiliations:** 1 Department of Urology, Jiangyin People’s Hospital, the Jiangyin Clinical College of Xuzhou Medical University, Jiangyin, Jiangsu, China; 2 Department of Urology, The Third Affiliated Hospital of Soochow University, Changzhou, Jiangsu, China; 3 Department of Urology, The First People’s Hospital of Changzhou, Changzhou, Jiangsu, China; 4 Department of Urology, The First Affiliated Hospital of Anhui Medical University, Hefei, Anhui, China; 5 Department of Urology, The Second Affiliated Hospital of Anhui Medical University, Hefei, China

**Keywords:** arteriogenic erectile dysfunction, biomarker, endothelial dysfunction, inflammation, neutrophil percentage-to-albumin ratio

## Abstract

**Background:**

The neutrophil percentage-to-albumin ratio (NPAR) is a novel composite marker reflecting both inflammatory and nutritional status. However, its association with arteriogenic erectile dysfunction (AED) has not been previously investigated. This study aimed to evaluate the relationship between NPAR and AED and to determine its diagnostic performance compared with conventional hematologic parameters.

**Methods:**

A total of 265 men were enrolled from three hospitals between April 2023 and August 2025, including 115 with AED, 53 with venogenic ED (VED), and 97 healthy controls. Erectile function was assessed by the five-item International Index of Erectile Function (IIEF-5), nocturnal penile tumescence and rigidity (NPTR) test and color duplex Doppler ultrasonography (CDDU) was used to classify ED subtypes. Laboratory parameters, including white blood cell (WBC) count, neutrophil percentage (NP), albumin, and NPAR, were measured. Group differences were analyzed using ANOVA or the Kruskal–Wallis test, and independent predictors of AED were identified using multivariate logistic regression. Diagnostic performance was evaluated using receiver operating characteristic (ROC) curve analysis. Subgroup analyses were conducted to assess robustness.

**Results:**

Compared with controls, individuals with AED exhibited significantly higher WBC levels (5.97 ± 1.13 vs. 5.45 ± 0.90 × 10^9^/L; P < 0.05). NPAR values were markedly elevated in the AED group (16.31 [6.36]) relative to both VED (12.65 [5.57]) and controls (12.61 [7.05]) (P < 0.001). In the fully adjusted multivariate model, NPAR remained independently associated with AED (OR = 1.258; 95% CI: 1.159–1.362; P < 0.001), as did WBC (OR = 1.984; P < 0.001). ROC analysis revealed that NPAR had the greatest discriminative ability among all hematologic markers (AUC = 0.742; 95% CI: 0.676–0.809), with an optimal cut-off of 15.046 (sensitivity = 0.652; specificity = 0.753), outperforming WBC, NP, albumin, C-reactive protein (CRP), and neutrophils/lymphocytes ratio (NLR).

**Conclusions:**

Elevated NPAR levels were significantly associated with AED. Compared with WBC and its individual components, NPAR exhibited superior discriminative ability for identifying AED. These findings suggest that NPAR may serve as a convenient and cost-effective hematologic parameter for assessing vascular risk in men with ED, although prospective studies are needed to validate this association.

## Introduction

Erectile dysfunction (ED) is defined as the persistent inability to achieve or maintain an erection sufficient for satisfactory sexual performance (1). It is one of the most common male sexual disorders, currently affecting approximately 150 million men worldwide, and this number is projected to exceed 300 million by 2025 (2). The prevalence of ED increases progressively with age and is strongly associated with multiple risk factors, including cardiovascular disease (CVD), metabolic syndrome, diabetes, and lifestyle habits such as smoking and physical inactivity (3–6). It is estimated that approximately 25%–70% of ED cases can be attributed to vascular causes, collectively referred to as vasculogenic erectile dysfunction (7), which encompasses arteriogenic (AED), venogenic (VED), and mixed forms (8). Among these, arteriogenic ED (AED) represents the predominant subtype, primarily resulting from structural or functional impairment of penile arteries (9). Clinical and epidemiological studies have identified several major risk factors for vasculogenic ED, including hypertension, diabetes, and dyslipidemia, all of which contribute to endothelial dysfunction within the penile vasculature (10).

Given the high prevalence of ED and its profound impact on quality of life, elucidating its underlying mechanisms and identifying novel predictive biomarkers have become major research priorities. Among the proposed mechanisms contributing to ED, inflammation plays a pivotal role. Chronic low-grade inflammation is closely linked to endothelial dysfunction, a key component in the pathogenesis of vasculogenic ED (11). The inflammatory process disrupts nitric oxide (NO) synthesis and bioavailability, leading to impaired vasodilation and reduced penile blood flow (12). Numerous studies have highlighted the association between inflammatory markers and ED. For instance, C-reactive protein (CRP), a widely recognized indicator of systemic inflammation, has been consistently correlated with ED severity (13). Similarly, hematologic ratios such as the neutrophil-to-lymphocyte ratio (NLR) and the platelet-to-lymphocyte ratio (PLR) have emerged as important predictors of ED, reflecting the balance between pro-inflammatory and anti-inflammatory pathways (14). Although growing evidence supports a strong link between inflammation and erectile dysfunction, the search for more comprehensive and integrative biomarkers that capture both inflammatory and metabolic components remain ongoing.

In recent years, the neutrophil percentage-to-albumin ratio (NPAR) has attracted increasing attention as a novel composite indicator reflecting both inflammatory and nutritional status. As classic cellular effectors, neutrophils play a central role in regulating inflammatory responses and mediating tissue injury during immune activation (15). Conversely, serum albumin acts as a key modulator of anti-inflammatory, antioxidant, and anticoagulant activity, and is inversely correlated with systemic inflammation (16, 17). Hypoalbuminemia also reflects poor nutritional status, which has been associated with adverse outcomes across a variety of chronic conditions (18). Recent studies have demonstrated that elevated NPAR levels are strongly associated with increased mortality in chronic obstructive pulmonary disease, CVD, and malignancies (19–21). By integrating both inflammatory and nutritional information, NPAR provides a more comprehensive reflection of systemic homeostasis and may serve as a valuable prognostic biomarker for disease outcomes.

However, to date, no study has examined the potential relationship between NPAR and ED, particularly AED—a subtype closely linked to systemic vascular and inflammatory processes. Given that NPAR simultaneously reflects both inflammatory activation and nutritional imbalance, it may provide a more integrated perspective on the pathophysiological mechanisms underlying AED. We therefore designed this study to investigate the association between NPAR and AED, and to evaluate whether NPAR could serve as an independent predictor and diagnostic biomarker for AED.

## Methods

### Study participants

From April 2023 to August 2025, we consecutively recruited male patients aged 18–65 years who attended the Urology/Andrology outpatient clinics of the Third Affiliated Hospital of Soochow University, Jiangyin People’s Hospital, and the Second Affiliated Hospital of Anhui Medical University with a primary diagnosis of ED. Each participant, including both patients and healthy controls, underwent a detailed clinical evaluation conducted by experienced andrology specialists using a pre-designed structured questionnaire. The questionnaire covered medical history, sexual history, comorbidities, and medication use. Erectile function was assessed using the five-item International Index of Erectile Function (IIEF-5), a score ≤21 was used to diagnose ED. Eligible participants reported regular sexual activity (≥1 time per week) with a stable female partner for at least six months, and a documented duration of ED symptoms of at least six months.

Exclusion criteria included: a history of pelvic or perineal surgery; endocrine disorders such as hypothyroidism, hyperthyroidism, androgen deficiency, or hyperprolactinemia; inflammatory diseases such as rheumatoid arthritis or osteoarthritis; neurological diseases; chronic alcoholism; and current use of medications known to affect sexual function or inflammatory status, including antipsychotics, antidepressants, or phosphodiesterase type 5 inhibitors; and any known renal disease, including chronic kidney disease, documented renal impairment, or persistent proteinuria/albuminuria. It should be noted that CVD was defined as a prior diagnosis of hypertension, coronary artery disease, myocardial infarction, stable angina, or peripheral arterial disease. Medication history was recorded for all participants. Patients taking drugs known to negatively affect erectile function—such as β-blockers (propranolol, metoprolol) or thiazide diuretics—were excluded. Antihypertensive agents used by included participants primarily consisted of ACE inhibitors, ARBs, or calcium channel blockers.

The control group was recruited from the health examination centers of the participating hospitals and met the same inclusion and exclusion criteria. These individuals had no symptoms suggestive of ED and exhibited normal erectile function, defined as an IIEF-5 score between 22 and 25 prior to enrollment. The study protocol was approved by the Institutional Ethics Committees of all participating centers, and written informed consent was obtained from all participants before enrollment.

### Hematologic measurements

After an overnight fast of at least 8 hours, venous blood samples were collected from all participants between 7:00 and 9:00 a.m. to minimize the influence of circadian variations in hormone and biochemical levels. Routine hematologic and biochemical analyses were performed in the clinical laboratories of each participating hospital using standardized procedures and quality control systems.

Complete blood counts (CBCs) were determined using an automated hematology analyzer (Sysmex XN-900, Sysmex Corporation, Kobe, Japan). Parameters including white blood cell (WBC) count and neutrophil percentage (NP) were recorded. Serum albumin, total cholesterol (TC), triglycerides (TG) were measured by direct assays and fasting blood glucose (FBG) levels were measured using the glucose oxidase method. C-reactive protein (CRP), serum total testosterone (TT) and sex hormone binding globulin (SHBG) was quantified by measured with chemiluminescent immunoassay (CLIA). Calculated free testosterone (FT) was derived using the Vermeulen equation based on TT, SHBG (22). The NPAR was calculated using the following formula: NPAR=Neutrophil percentage (%)/Albumin (g/dL). The neutrophils/lymphocytes ratio (NLR) was calculated using the following formula: NLR= neutrophils/lymphocytes.

All measurements were performed by trained laboratory technicians blinded to participants’ clinical information, and internal calibration and external quality control were routinely conducted to ensure data reliability.

### Nocturnal penile tumescence and rigidity test

All enrolled ED patients underwent continuous NPTR monitoring over two consecutive nights using the RigiScan™ device (GOTOP Inc., USA). Prior to the test, participants were instructed to avoid alcohol, caffeine, and other substances that could interfere with sleep, and to void their bladder before sleep. Monitoring took place from 22:00 to 08:00 each night.

The following NPTR parameters were recorded for analysis: base and tip rigidity, tumescence, event count, and the duration of the longest erection event. The NPTR results were classified as normal if one of the following criteria was met: (i) at least three erection events lasting ≥10 minutes in a single night with tip rigidity ≥70%, or (ii) penile circumference increase of ≥3 cm at the base or ≥2 cm at the tip. If neither of these criteria were met, the NPTR was considered abnormal (23).

### Color duplex Doppler ultrasonography test

ED patients with abnormal NPTR results subsequently underwent CDDU to further determine the etiology of ED. Prior to the procedure, all participants provided written informed consent after receiving detailed information about the potential risks of the test, including pain, infection, and prolonged erection, which are primarily associated with intra-cavernosal injection (ICI) of vasoactive agents.

Following the intra-cavernosal injection of 20 μg alprostadil (Caverject^®^, Pfizer, New York, USA), penile Doppler ultrasonography was performed by experienced radiologists using the Aixplorer™ ultrasound system (Supersonic Imagine S.A., Aix-en-Provence, France). The examination included measurement of peak systolic velocity (PSV), end-diastolic velocity (EDV) and acceleration time (AT) in the bilateral cavernous arteries. These parameters were recorded initially in the flaccid state and subsequently every 5 minutes for 25 minutes after ICI administration. Using these blood-flow velocities, the resistive index (RI) representing the venous leakage component can be calculated by: RI = (PSV - EDV)/PSV.

ED subtypes were classified using the following criteria: AED: PSV < 35 cm/s and EDV < 5 cm/s, or AT > 110 ms (24); VED: PSV ≥ 35 cm/s and EDV > 5 cm/s, with RI persistently< 0.6; Mixed vascular ED: PSV < 35 cm/s, EDV > 5 cm/s,and RI < 0.6; Non-vascular ED: PSV ≥ 35 cm/s, EDV < 5 cm/s, and RI > 0.8 (25, 26). Because the principal focus of this study was the assessment of AED, the diagnostic criteria for AED were prioritized in the vascular classification framework.

### Statistical analysis

All statistical analyses were performed using IBM SPSS Statistics version 26.0 (IBM Corp., Armonk, NY, USA) and GraphPad Prism version 9.0 (GraphPad Software, San Diego, CA, USA). Continuous variables were tested for normality using the Shapiro-Wilk test. Data are expressed as mean ± standard deviation (SD) for normally distributed variables or as median (interquartile range, IQR) for non-normally distributed variables. Categorical variables were presented as numbers and percentages. Comparisons among the AED, VED, and control groups were performed using one-way analysis of variance (ANOVA) for normally distributed data. The Kruskal–Wallis test for non-normally distributed data, and if there was a statistically significant difference, the Mann-Whitney U test was used for between-group comparisons. Chi-square tests were used to analyze categorical variables. To identify independent factors associated with AED, some related variables (age, BMI, smoking status, CVD, diabetes, WBC, FBG, TC, TG, TT, calculated FT and NPAR) were entered into a multivariate logistic regression model. Odds ratios (ORs) and 95% confidence intervals (CIs) were calculated. Sensitivity analyses using NPAR quartiles were also performed. Subgroup analyses were conducted according to age (<35 vs ≥35 years), smoking status, and CVD.

The diagnostic performance of NPAR and other hematologic parameters (WBC, NP, albumin, CRP, and NLR) in predicting AED was assessed using receiver operating characteristic (ROC) curve analysis. The area under the curve (AUC), optimal cut-off values, sensitivity, and specificity were determined according to the Youden index. A two-tailed P value < 0.05 was considered statistically significant for all analyses.

## Results

### Baseline characteristics of the study population

**Figure 1:**
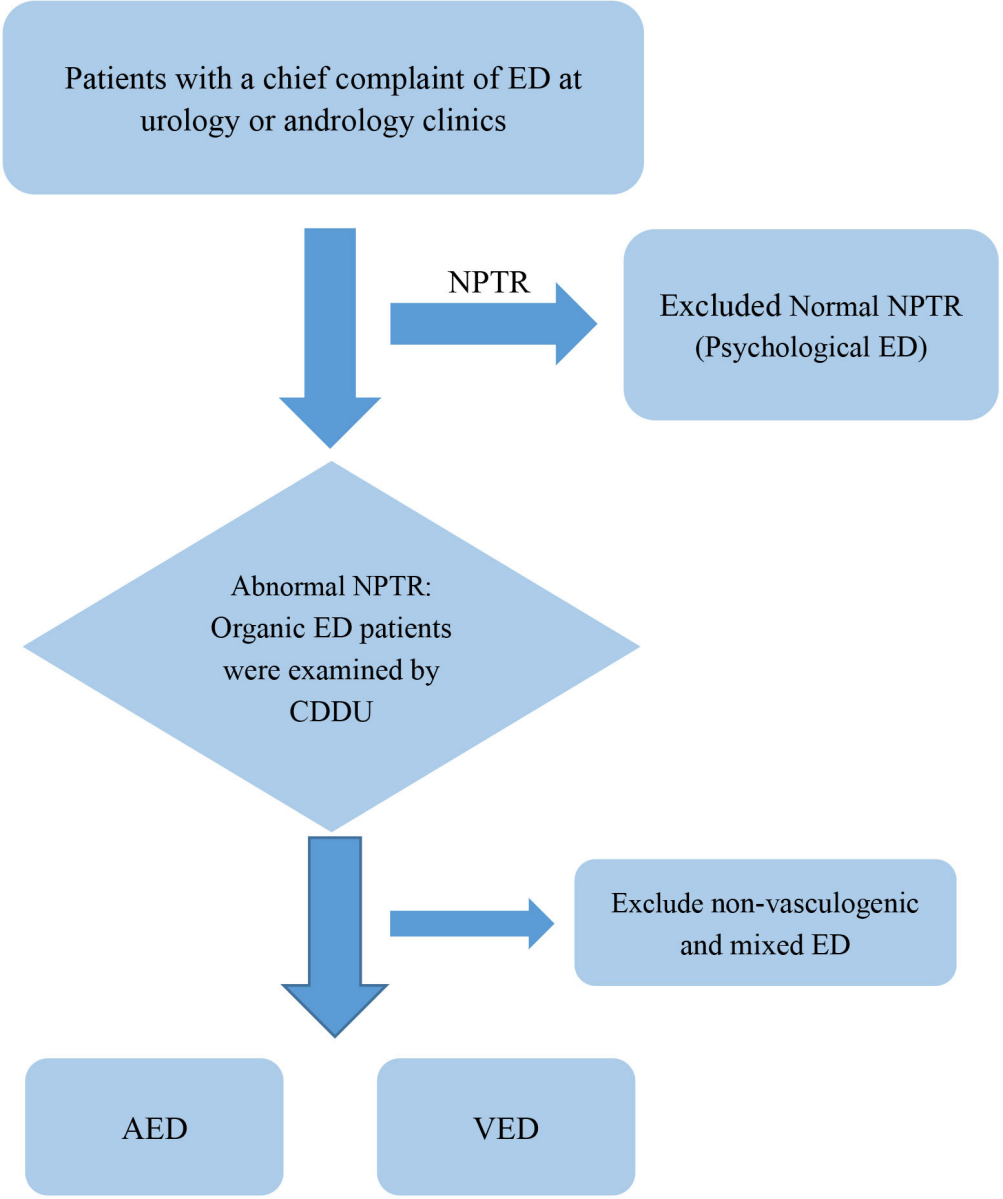
Flowchart of patient selection and classification.

A total of 265 participants were included: 115 with AED, 53 with VED, and 97 healthy controls (**Figure 1**). As shown in **Table 1**, there were no significant differences in age, BMI, smoking, or regular exercise across the groups (all P > 0.05). The AED group had a significantly higher prevalence of CVD compared to the control group (33.04% vs. 19.59%, P = 0.039).

**Table 1:** Demographic and clinical characteristics.

Variables	VED (n=53)	AED (n=115)	Control (n=97)	P
Age (years)	34.92 ± 11.02	36.19 ± 6.75	34.62 ± 6.44	0.300 **^c^**
BMI (kg/m^2^)	23.52 ± 3.20	23.82 ± 2.34	23.60 ± 1.60	0.684 **^c^**
Personal history				
Smoking, n (%)	20 (37.74)	43 (37.39)	31 (31.96)	0.661
Regular exercise, n (%)	14 (26.42)	30 (26.09)	30 (30.93)	0.709
Hematologic parameters				
WBC (10^9^/L)	5.65 ± 1.26	5.97 ± 1.13 **^b^**	5.45 ± 0.90	**0.003 ^c^**
NP, % *	53.46 (19.47) **^a^**	60.82 (25.87) **^b^**	52.47 (33.10)	**0.006 ^d^**
Albumin, g/dl	4.19 ± 0.48	4.04 ± 0.49	4.22 ± 0.47	0.052 **^c^**
NPAR *	12.65 (5.57) **^a^**	16.31 (6.36) **^b^**	12.61 (7.05)	**<0.001**
FBG (mmol/L) *	5.19 (0.43)	5.30 (0.69) **^b^**	5.01 (0.72)	0.053 **^d^**
TC (mmol/L) *	3.67 (0.91)	4.00 (0.83) **^b^**	3.58 (0.90)	**0.025 ^d^**
TG (mmol/L)	1.27 ± 0.57	1.42 ± 0.46	1.30 ± 0.35	0.065 **^c^**
TT (nmol/L)	17.57 ± 5.05	17.34 ± 4.07	18.13 ± 4.13	0.402 **^c^**
Calculated FT (pmol/L)	351.66 ± 99.13	338.11 ± 89.24	361.73 ± 78.48	0.127 **^c^**
CVD, n (%)	10 (18.87)	38 (33.04)	19 (19.59)	**0.039**
Diabetes, n (%)	6 (11.32)	18 (15.65)	12 (12.37)	0.680

Values are percentages or mean ± SD unless noted otherwise. *Values are presented as median with interquartile range. Regular exercise: Twice a week, at least 30 minutes each time.

ED, erectile dysfunction; VED, venous erectile dysfunction; AED, arteriogenic erectile dysfunction; BMI, Body Mass Index; WBC, white blood cells; NP, neutrophil percentage; NPAR, neutrophil percentage-to-albumin ratio; FBG, fasting blood glucose; TC, Total Cholesterol; TG, Triglyceride; TT, Total Testosterone; FT, free testosterone; NPAR, neutrophil percentage-to-albumin ratio; CVD, cardiovascular disease; P values are based on chi-square test unless noted otherwise. Bold indicates P <.05. a P <.05 vs AED; ^b^ P <.05 vs Control. ^c^ One-way analysis of variance. ^d^ Kruskal-Wallis test.

Hematologic parameters showed significant differences between groups. WBC was significantly higher in the AED group (5.97 ± 1.13 × 10^9^/L) compared to the control group (5.45 ± 0.90 × 10^9^/L, P <0.05). Similarly, NPAR was significantly higher in the AED group (16.31 (6.36)) compared to VED group (12.65 (5.57)) and control group (12.61 (7.05)) (P <0.001). TC was significantly higher in the AED group (4.00 ± 0.83 mmol/L) compared to the control group (3.58 ± 0.90 mmol/L, P = 0.025). No significant differences were observed in TG (P = 0.065).

### Association between NPAR and AED

**Figure 2:**
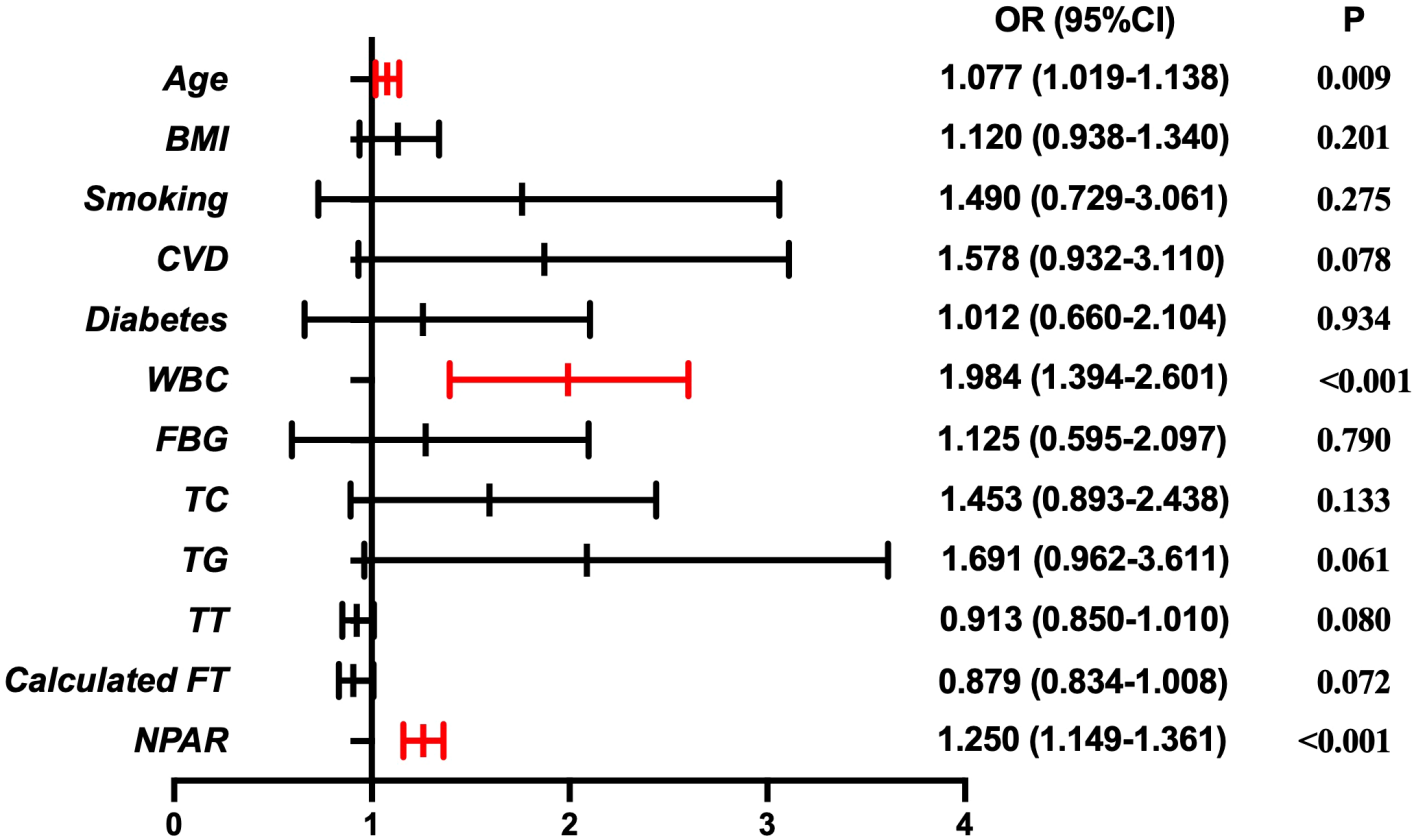
Multivariate logistic regression analysis for predictors of arteriogenic erectile dysfunction (AED). Forest plot illustrating the odds ratios (ORs) and 95% confidence intervals (CIs) for variables included in the multivariate logistic regression model.

**Table 2** presents the results of the multivariate logistic regression analysis to identify independent predictors of AED. The analysis showed that age was significantly associated with an increased risk of AED, with an OR of 1.077 (95% CI: 1.019–1.138, P = 0.009), indicating that older age increases the likelihood of AED. WBC count was also found to be a predictor of AED, with an OR of 1.984 (95% CI: 1.394–2.601, P < 0.001). Furthermore, NPAR was also identified as an independent predictor of AED, with an OR of 1.258 (95% CI: 1.159–1.362, P < 0.001). This finding reinforces the potential of NPAR as a useful biomarker for predicting AED, reflecting both inflammatory activation and nutritional imbalance. These findings are visually summarized in **Figure 2**, which illustrates the OR and 95% CI for each variable in predicting AED.

**Table 2:** Multivariate logistic regression analysis for AED (AED vs. control).

Variables	Multivariate logistic regression		
	OR	95%CI	P
Age	1.077	1.019-1.138	**0.009**
BMI	1.120	0.938-1.340	0.201
Smoking	1.490	0.729-3.061	0.275
CVD	1.578	0.932-3.110	0.078
Diabetes	1.012	0.660-2.104	0.934
WBC	1.984	1.394-2.601	**<0.001**
FBG	1.125	0.595-2.097	0.790
TC	1.453	0.893-2.438	0.123
TG	1.691	0.962-3.611	0.061
TT	0.913	0.850-1.010	0.080
Calculated FT	0.879	0.834-1.008	0.072
NPAR	1.258	1.159-1.362	**<0.001**

Multivariate logistic regression model included age, BMI, smoking status, CVD, diabetes, WBC, FBG, TC, TG, TT, calculated FT and NPAR.

ED, erectile dysfunction; AED, arteriogenic erectile dysfunction; BMI, Body Mass Index; CVD, cardiovascular disease; WBC, white blood cells; FBG, fasting blood glucose; TC, Total Cholesterol; TG, Triglyceride; TT, Total Testosterone; FT, free testosterone; NPAR, neutrophil percentage-to-albumin ratio. Bold indicates P < 0.05.

To further examine the dose–response relationship, NPAR was categorized into quartiles, and a fully adjusted regression model was repeated (**Supplementary Table S1**). The risk of AED increased progressively across NPAR quartiles, demonstrating a clear gradient: Q2 vs Q1: OR = 1.441 (95% CI 1.126–1.925; P = 0.011); Q3 vs Q1: OR = 2.430 (95% CI 2.130–3.042; P < 0.001); Q4 vs Q1: OR = 4.764 (95% CI 3.273–6.445; P < 0.001); This graded association provides strong evidence of a dose-dependent relationship between increasing NPAR and the likelihood of AED.

### Diagnostic performance of NPAR for identifying AED and subgroup analyses

**Figure 3:**
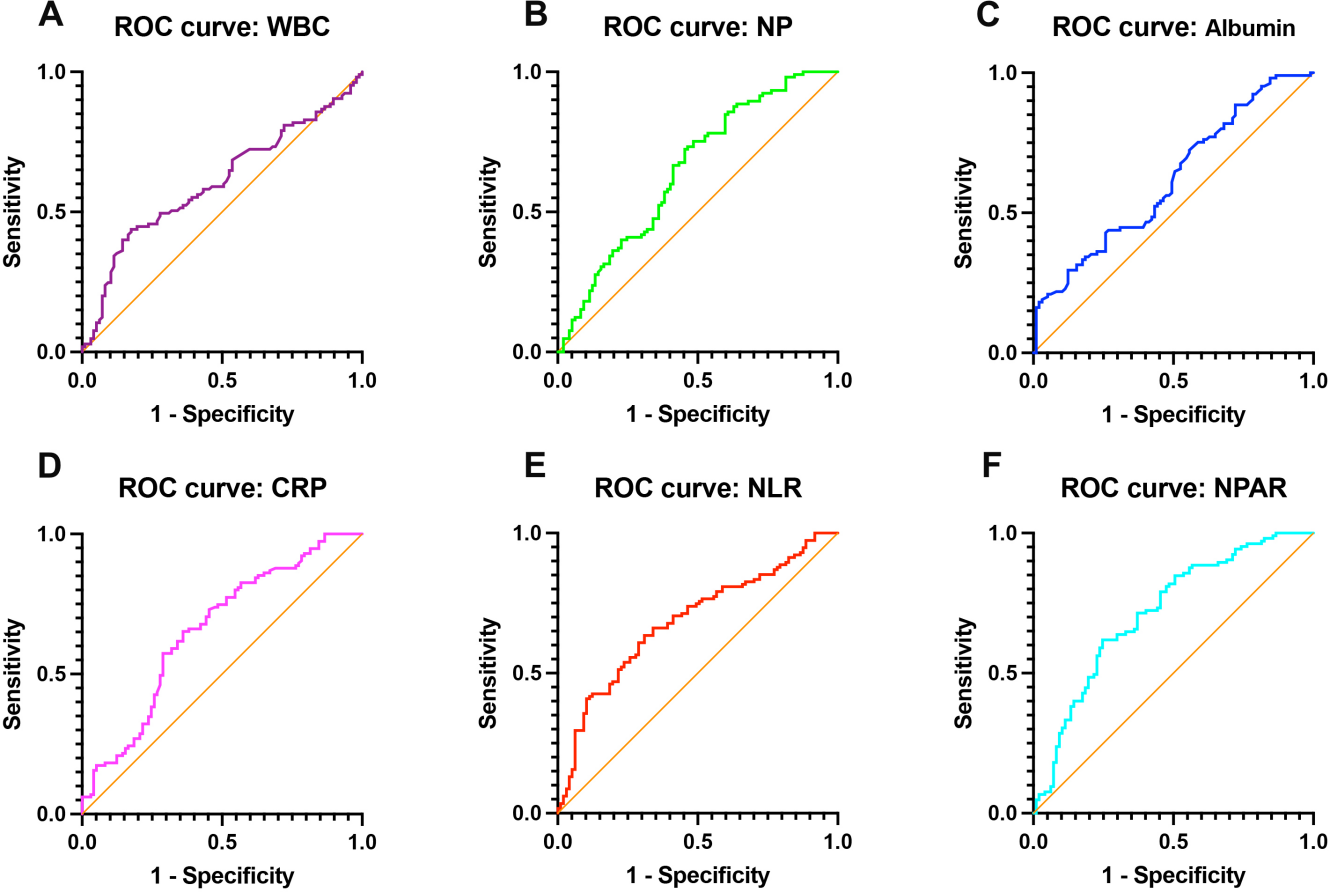
Receiver operating characteristic (ROC) curves of hematologic parameters for identifying arteriogenic erectile dysfunction (AED). ROC analysis comparing the diagnostic performance of: **(A)** white blood cell count (WBC); **(B)** neutrophil percentage (NP); **(C)** albumin; **(D)** C-reactive protein (CRP); **(E)** neutrophils/lymphocytes ratio (NLR); **(F)**neutrophil percentage-to-albumin ratio (NPAR) in detecting AED.

The diagnostic performance of hematologic indicators for identifying AED is summarized in **Table 3** and **Figure 3**. Among all parameters evaluated, NPAR demonstrated the highest diagnostic accuracy, with an area under the ROC curve (AUC) of 0.742 (95% CI: 0.676–0.809, P < 0.001). At an optimal cut-off value of 15.046, the sensitivity and specificity of NPAR were 0.652 and 0.753, respectively. In comparison, the AUCs for other hematologic markers were lower: WBC (AUC = 0.620, 95% CI: 0.545–0.696, P = 0.009), NP (AUC = 0.656, 95% CI: 0.582–0.730, P < 0.001), albumin (AUC = 0.633, 95% CI: 0.559–0.707, P = 0.001), CRP (AUC = 0.657, 95% CI: 0.583-0.732, P < 0.001), and NLR (AUC = 0.686, 95% CI: 0.614-0.757, P < 0.001). These results indicate that NPAR provides superior discriminatory ability for differentiating AED from non-arteriogenic cases. The ROC curve in **Figure 3** visually demonstrates that the curve for NPAR lies above those of WBC, NP, albumin, CRP, and NLR, confirming its better diagnostic performance.

**Table 3:** Diagnostic performance for identifying AED.

Indicator	Optimal cut-off	Specificity	Sensitivity	AUC (95%CI)	P value
WBC, 10^9^/L	6.185	0.825	0.461	0.620 (0.545-0.696)	0.009
NP, %	55.340	0.546	0.730	0.656 (0.582-0.730)	<0.001
Albumin, g/dl	3.865	0.470	0.742	0.633 (0.559-0.707)	0.001
CRP, mg/L	1.253	0.639	0.652	0.657 (0.583-0.732)	<0.001
NLR	1.998	0.691	0.635	0.686 (0.614-0.757)	<0.001
NPAR	15.046	0.753	0.652	0.742 (0.676-0.809)	<0.001

AED, arteriogenic erectile dysfunction; AUC, Area Under the Curve; CI, Confidence Interval; WBC, white blood cells; NP, neutrophil percentage; CRP, C-reactive protein; NLR, neutrophils/lymphocytes ratio; NPAR, neutrophil percentage-to-albumin ratio.

Subgroup analyses (**Supplementary Table S2**) demonstrated that the positive association between NPAR and AED was consistent across age groups, smoking status, and presence/absence of CVD: Age <35: OR = 1.163 (P = 0.002); Age ≥35: OR = 1.626 (P < 0.001); Non-smokers: OR = 1.252 (P < 0.001); Smokers: OR = 1.265 (P = 0.003); No CVD: OR = 1.271 (P = 0.006); With CVD: OR = 1.304 (P < 0.001). These findings support the robustness and biological plausibility of NPAR as an inflammatory–nutritional biomarker associated with AED.

## Discussion

In this cross-sectional study, we explored the relationship between the NPAR and AED. Our findings showed that NPAR levels were significantly higher in patients with AED compared with healthy controls. After adjusting for potential confounders, NPAR remained independently associated with the presence of AED, indicating that elevated NPAR values may reflect systemic inflammatory and nutritional disturbances that coexist with AED. Furthermore, ROC analysis demonstrated that NPAR had a greater discriminative ability for identifying AED than WBC or its individual components (NP and albumin) and other inflammation biomarkers (CRP and NLR). These results suggest that NPAR, as an integrated index, provides a more comprehensive reflection of the inflammatory and nutritional state related to vascular impairment in ED, supporting its potential utility as a convenient and informative hematologic parameter for clinical assessment.

Inflammation plays a pivotal role in the pathophysiology of ED. Among the extensively studied inflammatory markers, CRP is one of the most established indicators of systemic inflammation. Elevated CRP levels have been consistently associated with a higher prevalence of ED, as CRP is a known mediator of endothelial impairment, which is fundamental to normal erectile physiology. In addition, the NLR has been identified as a predictor of cardiovascular and inflammatory disorders, and recent studies have demonstrated a significant association between elevated NLR and ED (27). A higher NLR reflects a predominance of pro-inflammatory neutrophils, which may contribute to endothelial injury and vascular dysfunction. Similarly, the PLR has been proposed as another inflammation-related marker that reflects both inflammatory and thrombotic activity. Studies have shown that elevated PLR levels are associated with an increased risk of ED, suggesting that systemic inflammation and enhanced platelet activation may be involved in the pathogenesis of ED (28, 29). However, to date, no study has investigated the relationship between the NPAR and erectile dysfunction, particularly AED.

The NPAR integrates two critical biomarkers—NP and serum albumin level—and has increasingly been recognized as a reliable indicator of systemic inflammation in various chronic conditions. Previous studies have demonstrated that elevated NPAR levels are associated with adverse outcomes in cardiovascular diseases, including higher mortality and the progression of heart failure (30, 31). In addition, increased NPAR has been linked to a greater risk of chronic kidney disease (CKD). Given its composite nature, NPAR provides a comprehensive reflection of both pro-inflammatory and anti-inflammatory balance, combining the effects of neutrophil elevation and hypoalbuminemia. Our findings extend the existing evidence by showing that NPAR is independently associated with erectile dysfunction, particularly the arteriogenic subtype.

The potential mechanisms linking NPAR to AED may relate to the biological roles of its two components—neutrophils and albumin. Neutrophils are key mediators of the innate immune response and play a central role in the inflammatory cascade (32). Their activation results in the release of pro-inflammatory cytokines, reactive oxygen species (ROS), and other factors that contribute to endothelial dysfunction and vascular injury (33). Conversely, serum albumin is not merely a marker of nutritional status but also an important anti-inflammatory and antioxidant regulator. Lower albumin concentrations often indicate chronic inflammation and poor health status, and have been associated with adverse cardiovascular outcomes, endothelial impairment, and increased mortality (16, 17). The combined effect of elevated neutrophils and reduced albumin therefore offers an integrated perspective on systemic inflammation and vascular health. The strong association observed between NPAR and AED in our study may reflect this dual impact on endothelial function and vascular integrity.

Although the ROC analysis yielded a higher AUC for NPAR (0.742), these values remain insufficient for NPAR to function as an independent diagnostic tool. In interpreting the diagnostic performance of NPAR, it is important to emphasize that this biomarker is not intended to function as a standalone diagnostic or screening tool for AED. This is consistent with the biological nature of NPAR: as an inflammation- and nutrition-based composite index, it reflects systemic processes associated with endothelial impairment, but it cannot directly characterize penile hemodynamics. Rather than serving as a diagnostic test, the clinical value of NPAR lies in risk stratification. Elevated NPAR levels may suggest a greater inflammatory burden and a higher likelihood of vascular involvement among patients presenting with ED, thereby identifying individuals who may benefit from further cardiovascular or metabolic evaluation. This concept parallels the use of other inflammatory markers such as CRP or NLR, which do not offer high diagnostic accuracy but remain clinically valuable for estimating vascular risk. Notably, the robustness of this association across multiple clinically relevant subgroups—including different age categories, smoking statuses, and cardiovascular comorbidity profiles—suggests that NPAR may provide vascular risk information that is broadly applicable across diverse ED populations. The consistent findings across subgroups further strengthen the interpretation that NPAR reflects a systemic pathophysiological process rather than a phenomenon confined to a specific patient subset. Accordingly, NPAR should be viewed as a complementary biomarker—providing additional biological context to the hemodynamic information obtained through CDDU—rather than a substitute for Doppler ultrasound. Thus, while NPAR contributes meaningfully to the understanding of vascular risk in ED, its diagnostic and therapeutic implications should be interpreted within the broader framework of multifactorial ED assessment, and further studies are warranted to determine whether NPAR-guided strategies may improve clinical decision-making or long-term vascular outcomes.

Several limitations of this study should be acknowledged. First, the cross-sectional design precludes the establishment of a causal relationship between NPAR and AED. Second, although we adjusted for several major confounders, residual or unmeasured factors—such as diet, oxidative stress markers, and subclinical inflammation—may still have influenced the results. Third, the sample size was relatively modest and limited to patients recruited from specific clinical centers, which may restrict the generalizability of our findings. Fourth, cardiovascular outcomes and longitudinal follow-up data were unavailable; therefore, the current results cannot establish whether higher NPAR identifies AED patients at greater risk of future cardiovascular events. Another limitation relates to the demographic characteristics of the study population. Most participants in our cohort were relatively young and within the normal BMI range. This distribution reflects the typical attendance pattern of outpatient urology and andrology clinics in China, where older men or individuals with obesity are often reluctant to seek medical attention for sexual dysfunction due to cultural stigma and low health-seeking behavior. As a result, patients aged over 50–60 years—who comprise a large proportion of ED cases in the general population—were underrepresented. Therefore, the generalizability of our findings to older or overweight/obese populations may be limited. Future studies involving broader, community-based recruitment across diverse age and metabolic profiles are needed to validate these results. Finally, Renal dysfunction is an established risk factor for ED and may also influence systemic inflammatory and nutritional markers such as NPAR. In the present study, patients with any known kidney disease, including chronic kidney disease or persistent proteinuria/albuminuria, were excluded at baseline, and serum albumin levels were within the normal range across groups, making overt renal protein loss unlikely. However, creatinine and eGFR were not uniformly available across all centers, and subclinical renal impairment cannot be completely ruled out. Therefore, the absence of standardized renal function measurements represents a limitation and should be addressed in future studies. Despite these limitations, this study is, to our knowledge, the first to demonstrate a significant association between NPAR and AED. Our findings suggest that NPAR, a simple and cost-effective laboratory index combining inflammatory and nutritional information, may serve as a useful hematologic parameter for identifying individuals at higher vascular risk among ED patients. Future prospective and mechanistic studies are warranted to confirm these associations and to clarify the underlying biological pathways linking systemic inflammation, nutritional status, and vascular ED.

## Conclusion

In summary, this study demonstrated that the NPAR is significantly associated with AED. Elevated NPAR levels may reflect an imbalance between systemic inflammation and nutritional status that accompanies vascular impairment in ED. Compared with WBC and its individual components, NPAR showed superior discriminatory performance in identifying AED, highlighting its potential as a simple, inexpensive, and accessible hematologic indicator for clinical evaluation. Further prospective studies are warranted to validate these findings and to elucidate the underlying mechanisms linking NPAR to vascular erectile dysfunction.

## Data Availability

The original contributions presented in the study are included in the article/supplementary material. Further inquiries can be directed to the corresponding authors.
