# Supplementary material for: Neutrophil percentage-to-albumin ratio as a novel hematologic biomarker for predicting arteriogenic erectile dysfunction
Source: Front Endocrinol (Lausanne). 2026 Jan 19;16:1729618. doi: 10.3389/fendo.2025.1729618 (PMC12861870; doi:10.3389/fendo.2025.1729618)
Supplement: Supplementary file 1 [file Table1.docx]

**Table S1.** Association between NPAR and AED.

| Variable | Adjusted model | | |
| --- | --- | --- | --- |
|  | OR | 95%CI | P |
| NPAR (continuous) | 1.258 | 1.159-1.362 | **<0.001** |
| NPAR (Quartiles) |  |  |  |
| Q1 | Reference | Reference | Reference |
| Q2 | 1.441 | 1.126-1.925 | **0.011** |
| Q3 | 2.430 | 2.130-3.042 | **<0.001** |
| Q4 | 4.764 | 3.273-6.445 | **<0.001** |

**Statistical Analysis:**

Adjusted model: adjusted for age, BMI, smoking status, CVD, diabetes, WBC, FBG, TC, TG, TT, and calculated FT.

**Abbreviations:** ED, erectile dysfunction; AED, arteriogenic erectile dysfunction; BMI, Body Mass Index; CVD, cardiovascular disease; WBC, white blood cells; FBG, fasting blood glucose; TC, Total Cholesterol; TG, Triglyceride; TT, Total Testosterone; FT, free testosterone; NPAR, neutrophil percentage-to-albumin ratio; Q, quartiles. Bold indicates P < 0.05.

**Table S2.** Subgroup analyses of the association between NPAR and AED

| **Subgroup** | **OR** | **95%CI** | **P value** |
| --- | --- | --- | --- |
| Age group, % |  |  |  |
| <35y | 1.163 | 1.056-1.282 | 0.002 |
| >=35y | 1.626 | 1.303-2.030 | <0.001 |
| Smoking status, % |  |  |  |
| No | 1.252 | 1.120-1.401 | <0.001 |
| Yes | 1.265 | 1.087-1.472 | 0.003 |
| CVD, % |  |  |  |
| No | 1.271 | 1.152-1.402 | 0.006 |
| Yes | 1.304 | 1.150-1.519 | <0.001 |

**Statistical Analysis:** Adjusted model: adjusted for age, BMI, smoking status, CVD, diabetes, WBC, FBG, TC, TG, TT, and calculated FT. Subgroup analyses were conducted based on the fully adjusted multivariable model, with all covariates retained except for the subgroup-defining variable, which was removed from the model to avoid overadjustment.

**Abbreviations:** ED, erectile dysfunction; AED, arteriogenic erectile dysfunction; BMI, Body Mass Index; CVD, cardiovascular disease; WBC, white blood cells; FBG, fasting blood glucose; TC, Total Cholesterol; TG, Triglyceride; TT, Total Testosterone; FT, free testosterone; NPAR, neutrophil percentage-to-albumin ratio.
